# The *P*4_3_ enanti­omorph of Sr_2_As_2_O_7_

**DOI:** 10.1107/S1600536813031619

**Published:** 2013-11-23

**Authors:** Aicha Mbarek, Fadhila Edhokkar

**Affiliations:** a Laboratoire de Chimie Industrielle, Département de Génie des Matériaux, Ecole Nationale d’Ingénieurs de Sfax Université de Sfax, BP W3038, Sfax, Tunisia; b Laboratoire de l’Etat Solide, Faculté des Sciences, Université de Sfax, BP W3038, Sfax, Tunisia

## Abstract

The crystal structure of strontium diarsenate has been reinvestigated from single-crystal X-ray diffraction data. In contrast to the previous determinations of this structure [Weil *et al.* (2009 ▶). *Solid State Sci.* **11**, 2111–2117; Edhokkar *et al.* (2012 ▶). *Mater. Sci. Eng.*, **28**, 012017] and to all isotypic *A*_2_ *B*_2_O_7_ compounds that crystallize in the space group *P*4_1_, the current redetermination revealed the *P*4_3_ enanti­omorph of Sr_2_As_2_O_7_ with a purity of 96.3 (8)%. The crystal structure is made up from two eclipsed As_2_O_7_ diarsenate groups (symmetry 1) with characteristically longer As—O bridging bonds [1.756 (4)–1.781 (4) Å] than the terminal As—O bonds [1.636 (4)–1.679 (4) Å] and four Sr^2+^ sites with coordination numbers ranging from seven to nine. The building units are arranged in sheets parallel to (001).

## Related literature

The crystal structure of Sr_2_As_2_O_7_ has previously been refined from X-ray powder diffraction data (Weil *et al.*, 2009 ▶) in the space group *P*4_1_ and was later reinvestigated (Edhokkar *et al.*, 2012 ▶). For isotypic structures crystallizing in space group *P*4_1_, see: Baglio & Dann (1972 ▶); Webb (1966 ▶); Boudin *et al.* (1993 ▶); Müller-Bunz & Schleid (2000 ▶); Deng & Ibers (2005 ▶). For general structural features of the pyroarsenate anion, see: Weil & Stöger (2010 ▶).

## Experimental

### 

#### Crystal data

Sr_2_As_2_O_7_
*M* *_r_* = 437.08
Tetragonal, 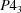

*a* = 7.1089 (1) Å
*c* = 25.6160 (4) Å
*V* = 1294.54 (4) Å^3^
*Z* = 8
Mo *K*α radiation
μ = 26.62 mm^−1^
*T* = 296 K
0.75 × 0.43 × 0.14 mm

#### Data collection

Bruker APEXII CCD diffractometer
Absorption correction: multi-scan (*SADABS*; Bruker, 2008 ▶) *T*_min_ = 0.448, *T*_max_ = 0.751
18524 measured reflections
4930 independent reflections
4593 reflections with *I* > 2σ(*I*)
*R*_int_ = 0.035

#### Refinement

*R*[*F* ^2^ > 2σ(*F* ^2^)] = 0.022
*wR*(*F* ^2^) = 0.044
*S* = 1.01
4930 reflections
201 parameters
1 restraint
Δρ_max_ = 0.92 e Å^−3^
Δρ_min_ = −1.34 e Å^−3^
Absolute structure: Flack (1983 ▶), 2400 Friedel pairs
Absolute structure parameter: 0.037 (8)

### 

Data collection: *APEX2* (Bruker, 2008 ▶); cell refinement: *SAINT* (Bruker, 2008 ▶); data reduction: *SAINT*; program(s) used to solve structure: *SHELXS97* (Sheldrick, 2008 ▶); program(s) used to refine structure: *SHELXL2013* (Sheldrick, 2008 ▶); molecular graphics: *DIAMOND* (Brandenburg, 1999 ▶) and *ORTEP-3 for Windows* (Farrugia, 2012 ▶); software used to prepare material for publication: *SHELXTL* (Sheldrick, 2008 ▶).

## Supplementary Material

Crystal structure: contains datablock(s) I, New_Global_Publ_Block. DOI: 10.1107/S1600536813031619/wm2770sup1.cif

Structure factors: contains datablock(s) I. DOI: 10.1107/S1600536813031619/wm2770Isup2.hkl

Additional supplementary materials:  crystallographic information; 3D view; checkCIF report

## Figures and Tables

**Fig. 1.**
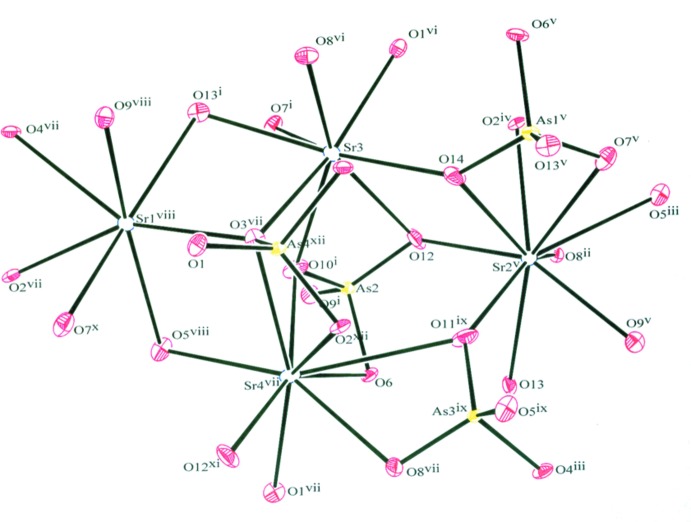
A view of a part of the structure of Sr2As2O7. Displacement ellipsoids are drawn at the 50% probability level. [Symmetry codes: (i) y, 1 - x, 1/4 + z; (ii) 1 - y, x, 3/4 + z; (iii) 1 - x, -y, 1/2 + z; (iv) 1 - y, -1 + x, 3/4 + z; (v) y, -x, 1/4 + z; (vi) -y, x, 3/4 + z; (vii) x, y, 1 + z; (viii) -x, 1 - y, 1/2 + z; (ix) -x, -y, 1/2 + z; (x) -1 + y, 1 - x, 1/4 + z; (xi) -1 + x, y, z; (xii) -y, -1 + x, 3/4 + z.]

**Fig. 2.**
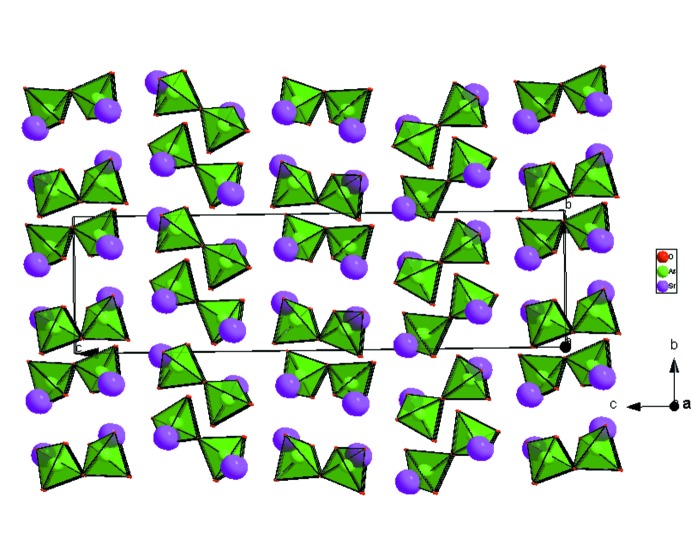
Projection along [100] of the Sr2As2O7 structure showing the stacking of (As2O7)4- sheets parallel to (001).

## supplementary crystallographic information

### 1. Comment

The structure of strontium diarsenate, Sr_2_As_2_O_7_, has previously been refined from X-ray powder diffraction data in the space group *P*4_1_ using the Rietveld method (Weil *et al.*, 2009). The structure was later reinvestigated from single-crystal X-ray diffraction data in the same space group (Edhokkar *et al.*, 2012) and is isotypic with Sr_2_V_2_O_7_ (Baglio & Dann, 1972), with Ce_2_Si_2_O_7_ and its homologous lighter *Ln*_2_Si_2_O_7_ lanthanides (*Ln* = La, Pr, Nd, Sm; Deng & Ibers, 2005), with the *A*-type structure of La_2_Si_2_O_7_ (Müller-Bunz & Schleid, 2000) and with all of the *β*-Ca_2_P_2_O_7_-type structures (Webb, 1966; Boudin *et al.*, 1993).

We took the opportunity to have obtained single crystals of good quality of Sr_2_As_2_O_7_ to improve the geometrical characteristics of this structure by a redetermination. Interestingly, it was found that in contrast to all above mentioned various *A*_2_*B*_2_O_7_ structures (space group *P*4_1_), the space group determined for the re-investigated material is *P*4_3_, with only a minor contribution of 3.7 (8)% for the *P*4_1_ enantiomorph. In comparison with the two previous studies, the precision in terms of bond lengths and angles is significantly higher for the current redetermination.

The structure of Sr_2_As_2_O_7_ is characterized by the presence of two independent eclipsed As_2_O_7_ diarsenate groups, both with site symmetry 1 (Fig. 1). The As—O bridging bonds are characteristically longer (Weil & Stöger, 2010) than the terminal As—O bonds. The brindging As—O bonds range from 1.756 (4) to 1.781 (4) Å, the terminal bonds from 1.636 (4) to 1.679 (4) Å. This trend is also observed in the closely related structures of *β*-Ca_2_P_2_O_7_ and Sr_2_V_2_O_7_ but to a lesser extent in La_2_Si_2_O_7_. This can be understood in terms of cationic repulsion since the *X*^5+^···*X*^5+^ (*X* = P, As, V) repulsion is stronger than that of Si^4+^··· Si^4+^. The As—O—As bridging angles, *viz.* 126.8 (2)° and 129.3 (2)°, are slightly greater than the corresponding V—O—V angles, 123.04° and 123.53°, in Sr_2_V_2_O_7_.

The crystal packing is based on discrete Sr^2+^ cations and isolated (As_2_O_7_)^4-^ anions arranged in sheets parallel to (001) (Fig. 2). The Sr^2+^ cations are divided into four independent atomic sites and exhibit coordination numbers from seven to nine, with irregular coordination polyhedra and Sr—O distances spreading over the range 2.458 (4) - 3.228 (5) Å.

### 2. Experimental

Single crystals of the title compound were synthesized in a solid state reaction by reacting As_2_O_5_ with SrCO_3_ in an alumina boat. A mixture of these reagents in the molar ratio 30:70 was used for the synthesis. The mixture was heated at 823 K for 24 h. After grinding, the reacting mixture was heated up to 1173 K and maintained at this temperature for 48 h. Then the mixture was cooled to room temperature by switching off the furnace power. Translucent single crystals of Sr_2_As_2_O_7_ were extracted from the batch.

### 3. Refinement

Reflections (0 0 4), (0 0 4), (0 1 2) and (0 1 1) were omitted from the refinement due to large differences between calculated and measured intensities. With regard to the anisotropic refinement of the atomic displacement parameters it should be mentioned that a first attempt carried out from the initial data collection routinely recorded using an exposure time of 10 s per frame resulted in some large ADP max/min ratios and either prolate or oblate displacement ellipsoids for some oxygen atoms. The corresponding value of θ_max_ for this data collection was 29.51°. Then a new data collection was carried out with an exposure time of 20 s per frame. The corresponding refinement lead to more homogeneous and acceptable values of the principal mean square atomic displacements *U*. For this data collection the θ_max_ value was also increased up to 33.22° and resulted in 4593 intensities with I>2σ(I) *versus* 3324 in the preceding data collection. The highest residual peak in the final difference Fourier map was located 0.70 Å from the Sr2 site and the deepest hole was located 0.65 Å from the As4 site.

### Crystal data

**Table:**

Sr_2_As_2_O_7_	*D*_x_ = 4.485 Mg m^−^^3^
*M**_r_* = 437.08	Mo *K*α radiation, λ = 0.71073 Å
Tetragonal, *P*4_3_	Cell parameters from 8103 reflections
*a* = 7.1089 (1) Å	θ = 3.3–33.2°
*c* = 25.6160 (4) Å	µ = 26.62 mm^−^^1^
*V* = 1294.54 (4) Å^3^	*T* = 296 K
*Z* = 8	Block, colourless
*F*(000) = 1584	0.75 × 0.43 × 0.14 mm

### Data collection

**Table:**

Bruker APEXII CCD diffractometer	4930 independent reflections
Radiation source: fine-focus sealed tube	4593 reflections with *I* > 2σ(*I*)
Detector resolution: 8.3333 pixels mm^-1^	*R*_int_ = 0.035
ω and φ scans	θ_max_ = 33.2°, θ_min_ = 2.9°
Absorption correction: multi-scan (*SADABS*; Bruker, 2008)	*h* = −10→10
*T*_min_ = 0.448, *T*_max_ = 0.751	*k* = −6→10
18524 measured reflections	*l* = −39→39

### Refinement

**Table:**

Refinement on *F*^2^	*w* = 1/[σ^2^(*F*_o_^2^) + (0.0055*P*)^2^] where *P* = (*F*_o_^2^ + 2*F*_c_^2^)/3
Least-squares matrix: full	(Δ/σ)_max_ = 0.021
*R*[*F*^2^ > 2σ(*F*^2^)] = 0.022	Δρ_max_ = 0.92 e Å^−^^3^
*wR*(*F*^2^) = 0.044	Δρ_min_ = −1.34 e Å^−^^3^
*S* = 1.01	Extinction correction: *SHELXL2013* (Sheldrick, 2008), Fc^*^=kFc[1+0.001xFc^2^λ^3^/sin(2θ)]^-1/4^
4930 reflections	Extinction coefficient: 0.00348 (16)
201 parameters	Absolute structure: Flack (1983), 2400 Friedel pairs
1 restraint	Absolute structure parameter: 0.037 (8)

### Special details

**Table:**

Geometry. All e.s.d.'s (except the e.s.d. in the dihedral angle between two l.s. planes) are estimated using the full covariance matrix. The cell e.s.d.'s are taken into account individually in the estimation of e.s.d.'s in distances, angles and torsion angles; correlations between e.s.d.'s in cell parameters are only used when they are defined by crystal symmetry. An approximate (isotropic) treatment of cell e.s.d.'s is used for estimating e.s.d.'s involving l.s. planes.
Refinement. Refined as a 2-component inversion twin.

### Fractional atomic coordinates and isotropic or equivalent isotropic displacement parameters (Å^2^)

**Table:**

	*x*	*y*	*z*	*U*_iso_*/*U*_eq_	
Sr1	0.26434 (7)	0.22687 (7)	0.42638 (2)	0.01083 (10)	
Sr2	0.02701 (7)	0.34822 (7)	0.57318 (2)	0.00890 (9)	
Sr3	0.15408 (7)	0.39584 (7)	0.92865 (2)	0.01120 (10)	
Sr4	0.37469 (7)	0.25769 (7)	0.06464 (2)	0.01125 (10)	
As1	0.14561 (7)	0.23414 (7)	0.69696 (2)	0.00774 (10)	
As2	0.22057 (7)	0.48276 (7)	0.79885 (2)	0.00756 (9)	
As3	0.18673 (7)	0.10525 (7)	0.29124 (2)	0.00678 (9)	
As4	1.24906 (7)	0.35443 (7)	0.19001 (2)	0.00640 (9)	
O1	0.4586 (5)	0.3299 (5)	−0.02876 (14)	0.0127 (7)	
O2	1.0936 (5)	0.3899 (6)	0.14240 (14)	0.0126 (7)	
O3	0.4187 (5)	0.2147 (5)	0.16739 (14)	0.0109 (7)	
O4	1.1085 (5)	0.2261 (5)	0.23449 (14)	0.0113 (7)	
O5	0.2999 (6)	0.2626 (5)	0.32782 (14)	0.0139 (7)	
O6	0.0777 (5)	0.3681 (6)	0.75140 (15)	0.0127 (7)	
O7	0.2725 (6)	0.3751 (6)	0.65898 (15)	0.0152 (8)	
O8	0.0556 (5)	0.3427 (6)	0.02051 (14)	0.0121 (7)	
O9	0.3251 (5)	0.3037 (6)	0.52047 (14)	0.0129 (7)	
O10	0.4811 (6)	0.0546 (6)	0.59334 (14)	0.0163 (8)	
O11	0.0026 (6)	0.0009 (7)	0.31632 (16)	0.0189 (8)	
O12	0.3813 (6)	0.3334 (6)	0.82115 (17)	0.0182 (8)	
O13	0.2817 (6)	0.0576 (5)	0.71776 (14)	0.0122 (7)	
O14	0.1680 (6)	0.0503 (6)	0.91819 (15)	0.0156 (8)	

### Atomic displacement parameters (Å^2^)

**Table:**

	*U*^11^	*U*^22^	*U*^33^	*U*^12^	*U*^13^	*U*^23^
Sr1	0.0132 (2)	0.0110 (2)	0.00828 (19)	0.00248 (18)	0.00247 (16)	0.00013 (16)
Sr2	0.0089 (2)	0.0093 (2)	0.00851 (18)	0.00103 (17)	0.00050 (15)	−0.00005 (16)
Sr3	0.0120 (2)	0.0120 (2)	0.00960 (19)	0.00213 (18)	−0.00190 (17)	0.00077 (16)
Sr4	0.0129 (2)	0.0132 (2)	0.00763 (18)	0.00355 (19)	−0.00066 (16)	0.00142 (16)
As1	0.0091 (2)	0.0073 (2)	0.0068 (2)	0.00129 (19)	−0.00043 (16)	−0.00053 (16)
As2	0.0083 (2)	0.0084 (2)	0.00595 (19)	−0.00026 (19)	−0.00020 (17)	0.00083 (17)
As3	0.0076 (2)	0.0081 (2)	0.00462 (19)	0.00012 (18)	0.00048 (16)	0.00111 (16)
As4	0.0073 (2)	0.0073 (2)	0.00457 (18)	0.00068 (18)	0.00009 (15)	−0.00018 (16)
O1	0.0133 (18)	0.0152 (19)	0.0096 (15)	0.0030 (15)	0.0030 (13)	−0.0028 (14)
O2	0.0128 (18)	0.0175 (19)	0.0075 (15)	−0.0007 (16)	−0.0048 (13)	0.0039 (14)
O3	0.0098 (17)	0.0108 (17)	0.0121 (16)	0.0030 (14)	0.0039 (13)	−0.0032 (13)
O4	0.0087 (17)	0.0170 (18)	0.0083 (15)	0.0000 (15)	−0.0006 (12)	0.0069 (13)
O5	0.020 (2)	0.0112 (18)	0.0101 (16)	−0.0031 (15)	−0.0064 (15)	−0.0013 (13)
O6	0.0123 (18)	0.0159 (18)	0.0098 (14)	0.0029 (15)	−0.0020 (13)	−0.0076 (13)
O7	0.0156 (19)	0.0155 (19)	0.0146 (17)	0.0006 (16)	0.0049 (14)	0.0042 (14)
O8	0.0122 (18)	0.0149 (19)	0.0092 (16)	−0.0041 (15)	−0.0006 (13)	0.0011 (13)
O9	0.0101 (18)	0.017 (2)	0.0114 (16)	0.0024 (15)	−0.0034 (13)	0.0018 (14)
O10	0.026 (2)	0.014 (2)	0.0090 (17)	0.0048 (17)	0.0049 (15)	0.0046 (13)
O11	0.0121 (19)	0.027 (2)	0.0178 (18)	−0.0041 (18)	0.0026 (15)	0.0114 (16)
O12	0.017 (2)	0.0113 (19)	0.026 (2)	0.0025 (16)	−0.0098 (16)	0.0051 (16)
O13	0.0158 (18)	0.0089 (17)	0.0120 (16)	0.0037 (15)	−0.0005 (14)	0.0017 (13)
O14	0.022 (2)	0.0133 (19)	0.0119 (17)	0.0013 (16)	−0.0051 (14)	0.0050 (14)

### Geometric parameters (Å, º)

**Table:**

Sr1—O9	2.509 (4)	As2—O12	1.661 (4)
Sr1—O5	2.550 (4)	As2—O6	1.781 (4)
Sr1—O13^i^	2.552 (4)	As2—Sr4^viii^	3.5090 (7)
Sr1—O3^ii^	2.555 (4)	As2—Sr1^ii^	3.6159 (7)
Sr1—O2^ii^	2.597 (4)	As2—Sr2^ii^	3.6547 (7)
Sr1—O7^iii^	2.622 (4)	As2—Sr2^iv^	3.7874 (7)
Sr1—O4^ii^	2.824 (4)	As2—Sr4^xiv^	3.8092 (7)
Sr1—As4^ii^	3.4611 (7)	As3—O11	1.636 (4)
Sr1—As3	3.6106 (6)	As3—O5	1.666 (4)
Sr1—As2^iii^	3.6159 (7)	As3—O8^iv^	1.679 (4)
Sr1—As1^i^	3.6288 (7)	As3—O4^xiii^	1.778 (4)
Sr1—As1^iii^	3.6500 (8)	As3—Sr2^iii^	3.4509 (7)
Sr2—O11^iv^	2.507 (4)	As3—Sr4^iv^	3.5007 (7)
Sr2—O9	2.533 (4)	As3—Sr3^xii^	3.7318 (7)
Sr2—O12^i^	2.573 (4)	As3—Sr4^ii^	3.7791 (7)
Sr2—O8^v^	2.644 (4)	As4—O1^xv^	1.654 (4)
Sr2—O2^vi^	2.710 (4)	As4—O2	1.665 (4)
Sr2—O14^i^	2.805 (4)	As4—O3^xvi^	1.666 (4)
Sr2—O13^i^	2.807 (4)	As4—O4	1.769 (4)
Sr2—O7	2.813 (4)	As4—Sr4^xvi^	3.4036 (7)
Sr2—O5^ii^	3.013 (4)	As4—Sr1^iii^	3.4610 (7)
Sr2—As1	3.3796 (7)	As4—Sr3^xvii^	3.6580 (7)
Sr2—As3^ii^	3.4509 (7)	As4—Sr4^xv^	3.7288 (7)
Sr2—As2^iii^	3.6547 (7)	As4—Sr3^xviii^	3.7738 (7)
Sr3—O10^ii^	2.458 (4)	As4—Sr1^xix^	3.7963 (7)
Sr3—O1^vii^	2.469 (4)	O1—As4^xx^	1.654 (4)
Sr3—O14	2.473 (4)	O1—Sr3^xxi^	2.469 (4)
Sr3—O8^vii^	2.484 (4)	O2—Sr1^iii^	2.597 (4)
Sr3—O13^ii^	2.594 (4)	O2—Sr2^xxii^	2.710 (4)
Sr3—O3^viii^	2.643 (4)	O2—Sr4^xvi^	2.974 (4)
Sr3—O7^ii^	2.878 (4)	O3—As4^xiii^	1.666 (4)
Sr3—O12	3.223 (5)	O3—Sr1^iii^	2.555 (4)
Sr3—As1^ii^	3.3422 (7)	O3—Sr3^xii^	2.643 (4)
Sr3—As2	3.4149 (7)	O4—As3^xvi^	1.778 (4)
Sr3—As4^ix^	3.6580 (7)	O4—Sr1^iii^	2.824 (4)
Sr3—As3^viii^	3.7317 (7)	O5—Sr4^ii^	2.618 (4)
Sr4—O1	2.519 (4)	O5—Sr2^iii^	3.013 (4)
Sr4—O10^x^	2.554 (4)	O6—Sr4^viii^	2.883 (4)
Sr4—O12^xi^	2.588 (4)	O7—Sr1^ii^	2.622 (4)
Sr4—O8	2.605 (4)	O7—Sr3^iii^	2.878 (4)
Sr4—O5^iii^	2.618 (4)	O8—As3^i^	1.679 (4)
Sr4—O3	2.668 (4)	O8—Sr3^xxi^	2.484 (4)
Sr4—O6^xii^	2.883 (4)	O8—Sr2^xxiii^	2.644 (4)
Sr4—O2^xiii^	2.974 (4)	O9—As2^iii^	1.656 (4)
Sr4—O11^i^	3.228 (5)	O10—As2^iii^	1.660 (4)
Sr4—As4^xiii^	3.4036 (7)	O10—Sr3^iii^	2.458 (4)
Sr4—As3^i^	3.5006 (7)	O10—Sr4^xxiv^	2.554 (4)
Sr4—As2^xii^	3.5091 (7)	O11—Sr2^i^	2.507 (4)
As1—O14^i^	1.644 (4)	O11—Sr4^iv^	3.228 (5)
As1—O7	1.663 (4)	O12—Sr2^iv^	2.573 (4)
As1—O13	1.672 (4)	O12—Sr4^xiv^	2.588 (4)
As1—O6	1.756 (4)	O13—Sr1^iv^	2.552 (4)
As1—Sr3^iii^	3.3422 (7)	O13—Sr3^iii^	2.594 (4)
As1—Sr1^iv^	3.6288 (7)	O13—Sr2^iv^	2.807 (4)
As1—Sr1^ii^	3.6501 (8)	O14—As1^iv^	1.644 (4)
As2—O9^ii^	1.656 (4)	O14—Sr2^iv^	2.805 (4)
As2—O10^ii^	1.660 (4)		
			
O9—Sr1—O5	155.84 (13)	O2^xiii^—Sr4—As4^xiii^	29.29 (7)
O9—Sr1—O13^i^	73.87 (12)	O11^i^—Sr4—As4^xiii^	83.33 (8)
O5—Sr1—O13^i^	119.01 (13)	O1—Sr4—As3^i^	95.60 (9)
O9—Sr1—O3^ii^	84.02 (12)	O10^x^—Sr4—As3^i^	108.37 (10)
O5—Sr1—O3^ii^	79.99 (12)	O12^xi^—Sr4—As3^i^	92.48 (9)
O13^i^—Sr1—O3^ii^	76.29 (12)	O8—Sr4—As3^i^	27.21 (8)
O9—Sr1—O2^ii^	116.98 (12)	O5^iii^—Sr4—As3^i^	176.99 (8)
O5—Sr1—O2^ii^	73.83 (12)	O3—Sr4—As3^i^	105.46 (8)
O13^i^—Sr1—O2^ii^	125.55 (13)	O6^xii^—Sr4—As3^i^	76.63 (8)
O3^ii^—Sr1—O2^ii^	151.94 (12)	O2^xiii^—Sr4—As3^i^	60.36 (7)
O9—Sr1—O7^iii^	88.17 (12)	O11^i^—Sr4—As3^i^	27.77 (7)
O5—Sr1—O7^iii^	73.88 (13)	As4^xiii^—Sr4—As3^i^	86.271 (16)
O13^i^—Sr1—O7^iii^	158.20 (13)	O1—Sr4—As2^xii^	92.07 (9)
O3^ii^—Sr1—O7^iii^	89.87 (12)	O10^x^—Sr4—As2^xii^	26.22 (8)
O2^ii^—Sr1—O7^iii^	73.47 (13)	O12^xi^—Sr4—As2^xii^	173.16 (9)
O9—Sr1—O4^ii^	72.18 (12)	O8—Sr4—As2^xii^	111.43 (9)
O5—Sr1—O4^ii^	127.99 (11)	O5^iii^—Sr4—As2^xii^	84.85 (9)
O13^i^—Sr1—O4^ii^	79.84 (12)	O3—Sr4—As2^xii^	88.69 (8)
O3^ii^—Sr1—O4^ii^	150.11 (11)	O6^xii^—Sr4—As2^xii^	30.39 (7)
O2^ii^—Sr1—O4^ii^	57.93 (10)	O2^xiii^—Sr4—As2^xii^	122.04 (8)
O7^iii^—Sr1—O4^ii^	106.75 (11)	O11^i^—Sr4—As2^xii^	68.79 (8)
O9—Sr1—As4^ii^	94.82 (9)	As4^xiii^—Sr4—As2^xii^	111.225 (17)
O5—Sr1—As4^ii^	100.31 (9)	As3^i^—Sr4—As2^xii^	93.097 (17)
O13^i^—Sr1—As4^ii^	105.86 (9)	O14^i^—As1—O7	111.7 (2)
O3^ii^—Sr1—As4^ii^	177.20 (8)	O14^i^—As1—O13	114.7 (2)
O2^ii^—Sr1—As4^ii^	27.46 (8)	O7—As1—O13	108.97 (19)
O7^iii^—Sr1—As4^ii^	87.54 (9)	O14^i^—As1—O6	106.16 (19)
O4^ii^—Sr1—As4^ii^	30.61 (7)	O7—As1—O6	106.7 (2)
O9—Sr1—As3	178.33 (9)	O13—As1—O6	108.23 (18)
O5—Sr1—As3	24.46 (9)	O14^i^—As1—Sr3^iii^	135.60 (14)
O13^i^—Sr1—As3	107.32 (8)	O7—As1—Sr3^iii^	59.43 (14)
O3^ii^—Sr1—As3	97.39 (8)	O13—As1—Sr3^iii^	49.64 (13)
O2^ii^—Sr1—As3	61.39 (8)	O6—As1—Sr3^iii^	118.15 (13)
O7^iii^—Sr1—As3	90.92 (9)	O14^i^—As1—Sr2	55.72 (14)
O4^ii^—Sr1—As3	106.77 (8)	O7—As1—Sr2	56.06 (15)
As4^ii^—Sr1—As3	83.737 (15)	O13—As1—Sr2	128.58 (13)
O9—Sr1—As2^iii^	23.60 (9)	O6—As1—Sr2	123.11 (13)
O5—Sr1—As2^iii^	144.18 (9)	Sr3^iii^—As1—Sr2	98.775 (17)
O13^i^—Sr1—As2^iii^	95.02 (8)	O14^i^—As1—Sr1^iv^	77.91 (15)
O3^ii^—Sr1—As2^iii^	99.15 (8)	O7—As1—Sr1^iv^	114.66 (14)
O2^ii^—Sr1—As2^iii^	96.26 (8)	O13—As1—Sr1^iv^	38.85 (13)
O7^iii^—Sr1—As2^iii^	70.30 (9)	O6—As1—Sr1^iv^	133.52 (14)
O4^ii^—Sr1—As2^iii^	65.18 (8)	Sr3^iii^—As1—Sr1^iv^	70.007 (15)
As4^ii^—Sr1—As2^iii^	78.954 (15)	Sr2—As1—Sr1^iv^	97.921 (16)
As3—Sr1—As2^iii^	154.799 (19)	O14^i^—As1—Sr1^ii^	110.41 (15)
O9—Sr1—As1^i^	93.63 (9)	O7—As1—Sr1^ii^	40.61 (14)
O5—Sr1—As1^i^	104.86 (9)	O13—As1—Sr1^ii^	133.44 (14)
O13^i^—Sr1—As1^i^	24.26 (8)	O6—As1—Sr1^ii^	68.19 (14)
O3^ii^—Sr1—As1^i^	92.02 (8)	Sr3^iii^—As1—Sr1^ii^	89.525 (17)
O2^ii^—Sr1—As1^i^	104.16 (9)	Sr2—As1—Sr1^ii^	70.709 (15)
O7^iii^—Sr1—As1^i^	177.52 (10)	Sr1^iv^—As1—Sr1^ii^	155.19 (2)
O4^ii^—Sr1—As1^i^	72.24 (8)	O9^ii^—As2—O10^ii^	115.3 (2)
As4^ii^—Sr1—As1^i^	90.599 (16)	O9^ii^—As2—O12	115.6 (2)
As3—Sr1—As1^i^	87.242 (16)	O10^ii^—As2—O12	110.6 (2)
As2^iii^—Sr1—As1^i^	110.953 (16)	O9^ii^—As2—O6	106.33 (19)
O9—Sr1—As1^iii^	74.19 (9)	O10^ii^—As2—O6	97.71 (19)
O5—Sr1—As1^iii^	93.54 (9)	O12—As2—O6	109.54 (19)
O13^i^—Sr1—As1^iii^	147.16 (8)	O9^ii^—As2—Sr3	128.75 (13)
O3^ii^—Sr1—As1^iii^	107.70 (8)	O10^ii^—As2—Sr3	42.37 (14)
O2^ii^—Sr1—As1^iii^	64.77 (9)	O12—As2—Sr3	69.19 (16)
O7^iii^—Sr1—As1^iii^	24.38 (8)	O6—As2—Sr3	120.18 (14)
O4^ii^—Sr1—As1^iii^	83.36 (8)	O9^ii^—As2—Sr4^viii^	125.22 (14)
As4^ii^—Sr1—As1^iii^	69.511 (15)	O10^ii^—As2—Sr4^viii^	42.80 (14)
As3—Sr1—As1^iii^	104.464 (16)	O12—As2—Sr4^viii^	119.14 (15)
As2^iii^—Sr1—As1^iii^	52.204 (13)	O6—As2—Sr4^viii^	54.98 (13)
As1^i^—Sr1—As1^iii^	155.19 (2)	Sr3—As2—Sr4^viii^	73.384 (15)
O11^iv^—Sr2—O9	84.28 (14)	O9^ii^—As2—Sr1^ii^	37.32 (13)
O11^iv^—Sr2—O12^i^	90.90 (14)	O10^ii^—As2—Sr1^ii^	121.84 (15)
O9—Sr2—O12^i^	146.49 (13)	O12—As2—Sr1^ii^	127.42 (16)
O11^iv^—Sr2—O8^v^	140.74 (13)	O6—As2—Sr1^ii^	69.06 (14)
O9—Sr2—O8^v^	91.02 (12)	Sr3—As2—Sr1^ii^	159.46 (2)
O12^i^—Sr2—O8^v^	72.09 (13)	Sr4^viii^—As2—Sr1^ii^	102.759 (16)
O11^iv^—Sr2—O2^vi^	134.89 (12)	O9^ii^—As2—Sr2^ii^	36.86 (13)
O9—Sr2—O2^vi^	134.38 (13)	O10^ii^—As2—Sr2^ii^	84.95 (15)
O12^i^—Sr2—O2^vi^	68.61 (12)	O12—As2—Sr2^ii^	112.34 (15)
O8^v^—Sr2—O2^vi^	72.11 (11)	O6—As2—Sr2^ii^	133.96 (13)
O11^iv^—Sr2—O14^i^	65.91 (12)	Sr3—As2—Sr2^ii^	92.341 (16)
O9—Sr2—O14^i^	124.71 (12)	Sr4^viii^—As2—Sr2^ii^	115.447 (18)
O12^i^—Sr2—O14^i^	82.20 (13)	Sr1^ii^—As2—Sr2^ii^	70.779 (14)
O8^v^—Sr2—O14^i^	141.14 (12)	O9^ii^—As2—Sr2^iv^	140.88 (14)
O2^vi^—Sr2—O14^i^	71.58 (12)	O10^ii^—As2—Sr2^iv^	101.84 (16)
O11^iv^—Sr2—O13^i^	75.47 (13)	O12—As2—Sr2^iv^	33.59 (15)
O9—Sr2—O13^i^	69.23 (11)	O6—As2—Sr2^iv^	79.11 (13)
O12^i^—Sr2—O13^i^	77.44 (12)	Sr3—As2—Sr2^iv^	72.528 (14)
O8^v^—Sr2—O13^i^	66.52 (11)	Sr4^viii^—As2—Sr2^iv^	90.211 (16)
O2^vi^—Sr2—O13^i^	132.64 (11)	Sr1^ii^—As2—Sr2^iv^	127.992 (17)
O14^i^—Sr2—O13^i^	135.72 (11)	Sr2^ii^—As2—Sr2^iv^	145.57 (2)
O11^iv^—Sr2—O7	99.69 (14)	O9^ii^—As2—Sr4^xiv^	82.28 (14)
O9—Sr2—O7	84.60 (11)	O10^ii^—As2—Sr4^xiv^	130.55 (14)
O12^i^—Sr2—O7	128.84 (12)	O12—As2—Sr4^xiv^	33.36 (15)
O8^v^—Sr2—O7	118.68 (12)	O6—As2—Sr4^xiv^	122.36 (12)
O2^vi^—Sr2—O7	68.80 (12)	Sr3—As2—Sr4^xiv^	89.707 (16)
O14^i^—Sr2—O7	58.30 (11)	Sr4^viii^—As2—Sr4^xiv^	152.50 (2)
O13^i^—Sr2—O7	153.66 (11)	Sr1^ii^—As2—Sr4^xiv^	100.390 (16)
O11^iv^—Sr2—O5^ii^	150.45 (13)	Sr2^ii^—As2—Sr4^xiv^	86.127 (15)
O9—Sr2—O5^ii^	70.10 (12)	Sr2^iv^—As2—Sr4^xiv^	63.658 (13)
O12^i^—Sr2—O5^ii^	118.58 (12)	O11—As3—O5	118.0 (2)
O8^v^—Sr2—O5^ii^	56.89 (11)	O11—As3—O8^iv^	110.1 (2)
O2^vi^—Sr2—O5^ii^	65.15 (12)	O5—As3—O8^iv^	108.43 (19)
O14^i^—Sr2—O5^ii^	116.99 (11)	O11—As3—O4^xiii^	106.86 (19)
O13^i^—Sr2—O5^ii^	107.28 (10)	O5—As3—O4^xiii^	106.65 (18)
O7—Sr2—O5^ii^	64.40 (11)	O8^iv^—As3—O4^xiii^	106.08 (17)
O11^iv^—Sr2—As1	81.11 (10)	O11—As3—Sr2^iii^	126.89 (15)
O9—Sr2—As1	105.15 (8)	O5—As3—Sr2^iii^	60.81 (14)
O12^i^—Sr2—As1	106.81 (10)	O8^iv^—As3—Sr2^iii^	48.13 (13)
O8^v^—Sr2—As1	137.18 (8)	O4^xiii^—As3—Sr2^iii^	124.85 (12)
O2^vi^—Sr2—As1	68.28 (8)	O11—As3—Sr4^iv^	66.80 (17)
O14^i^—Sr2—As1	28.97 (8)	O5—As3—Sr4^iv^	119.24 (13)
O13^i^—Sr2—As1	156.30 (8)	O8^iv^—As3—Sr4^iv^	45.18 (13)
O7—Sr2—As1	29.36 (8)	O4^xiii^—As3—Sr4^iv^	130.99 (13)
O5^ii^—Sr2—As1	91.35 (7)	Sr2^iii^—As3—Sr4^iv^	70.382 (15)
O11^iv^—Sr2—As3^ii^	161.51 (9)	O11—As3—Sr1	81.62 (15)
O9—Sr2—As3^ii^	81.87 (9)	O5—As3—Sr1	39.32 (14)
O12^i^—Sr2—As3^ii^	93.90 (10)	O8^iv^—As3—Sr1	111.43 (13)
O8^v^—Sr2—As3^ii^	28.22 (8)	O4^xiii^—As3—Sr1	135.74 (13)
O2^vi^—Sr2—As3^ii^	63.13 (8)	Sr2^iii^—As3—Sr1	70.419 (14)
O14^i^—Sr2—As3^ii^	132.45 (8)	Sr4^iv^—As3—Sr1	92.705 (15)
O13^i^—Sr2—As3^ii^	88.16 (8)	O11—As3—Sr3^xii^	113.46 (17)
O7—Sr2—As3^ii^	91.16 (8)	O5—As3—Sr3^xii^	125.02 (14)
O5^ii^—Sr2—As3^ii^	28.88 (7)	O8^iv^—As3—Sr3^xii^	32.45 (12)
As1—Sr2—As3^ii^	114.339 (17)	O4^xiii^—As3—Sr3^xii^	74.41 (13)
O11^iv^—Sr2—As2^iii^	79.15 (11)	Sr2^iii^—As3—Sr3^xii^	73.859 (14)
O9—Sr2—As2^iii^	23.09 (8)	Sr4^iv^—As3—Sr3^xii^	66.010 (14)
O12^i^—Sr2—As2^iii^	165.61 (9)	Sr1—As3—Sr3^xii^	142.980 (19)
O8^v^—Sr2—As2^iii^	109.34 (8)	O11—As3—Sr4^ii^	119.22 (17)
O2^vi^—Sr2—As2^iii^	125.72 (9)	O5—As3—Sr4^ii^	35.87 (13)
O14^i^—Sr2—As2^iii^	102.79 (8)	O8^iv^—As3—Sr4^ii^	128.93 (14)
O13^i^—Sr2—As2^iii^	89.91 (8)	O4^xiii^—As3—Sr4^ii^	72.47 (13)
O7—Sr2—As2^iii^	63.81 (8)	Sr2^iii^—As3—Sr4^ii^	89.553 (16)
O5^ii^—Sr2—As2^iii^	71.50 (8)	Sr4^iv^—As3—Sr4^ii^	155.10 (2)
As1—Sr2—As2^iii^	82.100 (15)	Sr1—As3—Sr4^ii^	66.002 (13)
As3^ii^—Sr2—As2^iii^	92.506 (17)	Sr3^xii^—As3—Sr4^ii^	123.551 (17)
O10^ii^—Sr3—O1^vii^	135.45 (13)	O1^xv^—As4—O2	117.59 (19)
O10^ii^—Sr3—O14	105.58 (13)	O1^xv^—As4—O3^xvi^	113.31 (19)
O1^vii^—Sr3—O14	79.88 (13)	O2—As4—O3^xvi^	108.41 (19)
O10^ii^—Sr3—O8^vii^	144.63 (13)	O1^xv^—As4—O4	107.41 (18)
O1^vii^—Sr3—O8^vii^	78.44 (12)	O2—As4—O4	100.09 (18)
O14—Sr3—O8^vii^	87.88 (13)	O3^xvi^—As4—O4	108.97 (18)
O10^ii^—Sr3—O13^ii^	87.30 (13)	O1^xv^—As4—Sr4^xvi^	121.83 (13)
O1^vii^—Sr3—O13^ii^	103.23 (12)	O2—As4—Sr4^xvi^	60.90 (13)
O14—Sr3—O13^ii^	158.47 (13)	O3^xvi^—As4—Sr4^xvi^	50.32 (13)
O8^vii^—Sr3—O13^ii^	72.18 (12)	O4—As4—Sr4^xvi^	130.67 (13)
O10^ii^—Sr3—O3^viii^	66.15 (12)	O1^xv^—As4—Sr1^iii^	122.48 (14)
O1^vii^—Sr3—O3^viii^	158.40 (12)	O2—As4—Sr1^iii^	46.00 (14)
O14—Sr3—O3^viii^	95.10 (13)	O3^xvi^—As4—Sr1^iii^	124.19 (13)
O8^vii^—Sr3—O3^viii^	80.40 (12)	O4—As4—Sr1^iii^	54.37 (12)
O13^ii^—Sr3—O3^viii^	74.08 (12)	Sr4^xvi^—As4—Sr1^iii^	97.122 (16)
O10^ii^—Sr3—O7^ii^	73.13 (13)	O1^xv^—As4—Sr3^xvii^	34.11 (13)
O1^vii^—Sr3—O7^ii^	75.77 (13)	O2—As4—Sr3^xvii^	89.22 (14)
O14—Sr3—O7^ii^	140.63 (13)	O3^xvi^—As4—Sr3^xvii^	109.59 (13)
O8^vii^—Sr3—O7^ii^	116.38 (12)	O4—As4—Sr3^xvii^	134.84 (13)
O13^ii^—Sr3—O7^ii^	59.20 (11)	Sr4^xvi^—As4—Sr3^xvii^	92.470 (16)
O3^viii^—Sr3—O7^ii^	118.18 (12)	Sr1^iii^—As4—Sr3^xvii^	116.588 (18)
O10^ii^—Sr3—O12	55.50 (11)	O1^xv^—As4—Sr4^xv^	33.42 (13)
O1^vii^—Sr3—O12	84.94 (11)	O2—As4—Sr4^xv^	124.04 (14)
O14—Sr3—O12	75.56 (12)	O3^xvi^—As4—Sr4^xv^	126.42 (13)
O8^vii^—Sr3—O12	158.38 (11)	O4—As4—Sr4^xv^	73.99 (13)
O13^ii^—Sr3—O12	125.74 (11)	Sr4^xvi^—As4—Sr4^xv^	155.21 (2)
O3^viii^—Sr3—O12	114.35 (11)	Sr1^iii^—As4—Sr4^xv^	101.419 (16)
O7^ii^—Sr3—O12	71.92 (11)	Sr3^xvii^—As4—Sr4^xv^	64.541 (13)
O10^ii^—Sr3—As1^ii^	80.01 (10)	O1^xv^—As4—Sr3^xviii^	135.30 (14)
O1^vii^—Sr3—As1^ii^	88.50 (9)	O2—As4—Sr3^xviii^	105.79 (14)
O14—Sr3—As1^ii^	167.75 (10)	O3^xvi^—As4—Sr3^xviii^	37.06 (13)
O8^vii^—Sr3—As1^ii^	93.63 (9)	O4—As4—Sr3^xviii^	73.24 (13)
O13^ii^—Sr3—As1^ii^	29.41 (8)	Sr4^xvi^—As4—Sr3^xviii^	70.193 (14)
O3^viii^—Sr3—As1^ii^	97.14 (8)	Sr1^iii^—As4—Sr3^xviii^	94.890 (16)
O7^ii^—Sr3—As1^ii^	29.83 (8)	Sr3^xvii^—As4—Sr3^xviii^	146.09 (2)
O12—Sr3—As1^ii^	99.72 (7)	Sr4^xv^—As4—Sr3^xviii^	123.790 (16)
O10^ii^—Sr3—As2	27.08 (9)	O1^xv^—As4—Sr1^xix^	80.74 (14)
O1^vii^—Sr3—As2	110.07 (8)	O2—As4—Sr1^xix^	127.56 (14)
O14—Sr3—As2	93.94 (9)	O3^xvi^—As4—Sr1^xix^	32.57 (13)
O8^vii^—Sr3—As2	171.48 (9)	O4—As4—Sr1^xix^	121.76 (12)
O13^ii^—Sr3—As2	104.64 (8)	Sr4^xvi^—As4—Sr1^xix^	67.769 (14)
O3^viii^—Sr3—As2	91.14 (8)	Sr1^iii^—As4—Sr1^xix^	156.76 (2)
O7^ii^—Sr3—As2	66.78 (8)	Sr3^xvii^—As4—Sr1^xix^	82.756 (15)
O12—Sr3—As2	28.80 (7)	Sr4^xv^—As4—Sr1^xix^	98.608 (16)
As1^ii^—Sr3—As2	86.359 (17)	Sr3^xviii^—As4—Sr1^xix^	63.894 (13)
O10^ii^—Sr3—As4^ix^	116.55 (9)	As4^xx^—O1—Sr3^xxi^	123.83 (18)
O1^vii^—Sr3—As4^ix^	22.06 (8)	As4^xx^—O1—Sr4	125.37 (19)
O14—Sr3—As4^ix^	71.77 (10)	Sr3^xxi^—O1—Sr4	104.52 (13)
O8^vii^—Sr3—As4^ix^	98.65 (9)	As4—O2—Sr1^iii^	106.54 (17)
O13^ii^—Sr3—As4^ix^	118.28 (8)	As4—O2—Sr2^xxii^	142.5 (2)
O3^viii^—Sr3—As4^ix^	166.86 (8)	Sr1^iii^—O2—Sr2^xxii^	100.24 (13)
O7^ii^—Sr3—As4^ix^	74.11 (8)	As4—O2—Sr4^xvi^	89.82 (15)
O12—Sr3—As4^ix^	63.25 (7)	Sr1^iii^—O2—Sr4^xvi^	134.85 (15)
As1^ii^—Sr3—As4^ix^	95.994 (17)	Sr2^xxii^—O2—Sr4^xvi^	89.51 (10)
As2—Sr3—As4^ix^	89.820 (16)	As4^xiii^—O3—Sr1^iii^	126.87 (19)
O10^ii^—Sr3—As3^viii^	133.38 (9)	As4^xiii^—O3—Sr3^xii^	120.60 (19)
O1^vii^—Sr3—As3^viii^	90.92 (9)	Sr1^iii^—O3—Sr3^xii^	100.82 (13)
O14—Sr3—As3^viii^	73.01 (9)	As4^xiii^—O3—Sr4	100.96 (17)
O8^vii^—Sr3—As3^viii^	21.27 (9)	Sr1^iii^—O3—Sr4	100.88 (12)
O13^ii^—Sr3—As3^viii^	85.58 (8)	Sr3^xii^—O3—Sr4	102.36 (12)
O3^viii^—Sr3—As3^viii^	67.59 (8)	As4—O4—As3^xvi^	126.8 (2)
O7^ii^—Sr3—As3^viii^	137.00 (8)	As4—O4—Sr1^iii^	95.02 (14)
O12—Sr3—As3^viii^	148.54 (7)	As3^xvi^—O4—Sr1^iii^	137.87 (18)
As1^ii^—Sr3—As3^viii^	111.362 (17)	As3—O5—Sr1	116.2 (2)
As2—Sr3—As3^viii^	153.25 (2)	As3—O5—Sr4^ii^	122.23 (19)
As4^ix^—Sr3—As3^viii^	107.264 (16)	Sr1—O5—Sr4^ii^	102.39 (13)
O1—Sr4—O10^x^	110.84 (12)	As3—O5—Sr2^iii^	90.31 (16)
O1—Sr4—O12^xi^	83.45 (13)	Sr1—O5—Sr2^iii^	93.78 (12)
O10^x^—Sr4—O12^xi^	152.75 (13)	Sr4^ii^—O5—Sr2^iii^	129.62 (15)
O1—Sr4—O8	75.33 (12)	As1—O6—As2	129.3 (2)
O10^x^—Sr4—O8	132.54 (13)	As1—O6—Sr4^viii^	133.0 (2)
O12^xi^—Sr4—O8	72.49 (13)	As2—O6—Sr4^viii^	94.63 (15)
O1—Sr4—O5^iii^	82.29 (12)	As1—O7—Sr1^ii^	115.01 (19)
O10^x^—Sr4—O5^iii^	70.56 (14)	As1—O7—Sr2	94.57 (18)
O12^xi^—Sr4—O5^iii^	89.41 (13)	Sr1^ii^—O7—Sr2	97.04 (13)
O8—Sr4—O5^iii^	152.48 (13)	As1—O7—Sr3^iii^	90.74 (16)
O1—Sr4—O3	158.86 (13)	Sr1^ii^—O7—Sr3^iii^	127.25 (16)
O10^x^—Sr4—O3	64.49 (11)	Sr2—O7—Sr3^iii^	127.44 (15)
O12^xi^—Sr4—O3	93.63 (12)	As3^i^—O8—Sr3^xxi^	126.29 (19)
O8—Sr4—O3	123.82 (11)	As3^i^—O8—Sr4	107.61 (17)
O5^iii^—Sr4—O3	76.73 (11)	Sr3^xxi^—O8—Sr4	101.60 (13)
O1—Sr4—O6^xii^	68.82 (11)	As3^i^—O8—Sr2^xxiii^	103.66 (17)
O10^x^—Sr4—O6^xii^	56.58 (11)	Sr3^xxi^—O8—Sr2^xxiii^	114.81 (14)
O12^xi^—Sr4—O6^xii^	148.69 (12)	Sr4—O8—Sr2^xxiii^	99.49 (12)
O8—Sr4—O6^xii^	86.22 (11)	As2^iii^—O9—Sr1	119.1 (2)
O5^iii^—Sr4—O6^xii^	100.56 (11)	As2^iii^—O9—Sr2	120.05 (18)
O3—Sr4—O6^xii^	117.48 (11)	Sr1—O9—Sr2	113.29 (14)
O1—Sr4—O2^xiii^	136.97 (11)	As2^iii^—O10—Sr3^iii^	110.6 (2)
O10^x^—Sr4—O2^xiii^	110.59 (11)	As2^iii^—O10—Sr4^xxiv^	110.98 (18)
O12^xi^—Sr4—O2^xiii^	64.36 (11)	Sr3^iii^—O10—Sr4^xxiv^	111.31 (15)
O8—Sr4—O2^xiii^	68.43 (11)	As3—O11—Sr2^i^	142.2 (2)
O5^iii^—Sr4—O2^xiii^	122.63 (11)	As3—O11—Sr4^iv^	85.43 (17)
O3—Sr4—O2^xiii^	56.92 (10)	Sr2^i^—O11—Sr4^iv^	128.41 (16)
O6^xii^—Sr4—O2^xiii^	128.82 (11)	As2—O12—Sr2^iv^	125.5 (2)
O1—Sr4—O11^i^	108.85 (12)	As2—O12—Sr4^xiv^	126.0 (2)
O10^x^—Sr4—O11^i^	80.77 (12)	Sr2^iv^—O12—Sr4^xiv^	101.82 (14)
O12^xi^—Sr4—O11^i^	117.54 (11)	As2—O12—Sr3	82.01 (16)
O8—Sr4—O11^i^	54.27 (11)	Sr2^iv^—O12—Sr3	94.25 (13)
O5^iii^—Sr4—O11^i^	151.33 (12)	Sr4^xiv^—O12—Sr3	122.43 (15)
O3—Sr4—O11^i^	91.11 (11)	As1—O13—Sr1^iv^	116.89 (19)
O6^xii^—Sr4—O11^i^	61.93 (11)	As1—O13—Sr3^iii^	100.95 (16)
O2^xiii^—Sr4—O11^i^	67.23 (11)	Sr1^iv^—O13—Sr3^iii^	102.21 (13)
O1—Sr4—As4^xiii^	156.53 (9)	As1—O13—Sr2^iv^	124.42 (18)
O10^x^—Sr4—As4^xiii^	90.53 (8)	Sr1^iv^—O13—Sr2^iv^	103.48 (12)
O12^xi^—Sr4—As4^xiii^	73.09 (9)	Sr3^iii^—O13—Sr2^iv^	106.22 (13)
O8—Sr4—As4^xiii^	97.70 (8)	As1^iv^—O14—Sr3	143.5 (2)
O5^iii^—Sr4—As4^xiii^	96.52 (8)	As1^iv^—O14—Sr2^iv^	95.30 (18)
O3—Sr4—As4^xiii^	28.72 (8)	Sr3—O14—Sr2^iv^	107.87 (14)
O6^xii^—Sr4—As4^xiii^	133.87 (8)		

Symmetry codes: (i) −*y*, *x*, *z*−1/4; (ii) *y*, −*x*+1, *z*+1/4; (iii) −*y*+1, *x*, *z*−1/4; (iv) *y*, −*x*, *z*+1/4; (v) −*x*, −*y*+1, *z*+1/2; (vi) −*x*+1, −*y*+1, *z*+1/2; (vii) *x*, *y*, *z*+1; (viii) −*y*, *x*, *z*+3/4; (ix) −*y*+1, *x*−1, *z*+3/4; (x) −*x*+1, −*y*, *z*−1/2; (xi) *y*, −*x*+1, *z*−3/4; (xii) *y*, −*x*, *z*−3/4; (xiii) *x*−1, *y*, *z*; (xiv) −*y*+1, *x*, *z*+3/4; (xv) *y*+1, −*x*+1, *z*+1/4; (xvi) *x*+1, *y*, *z*; (xvii) *y*+1, −*x*+1, *z*−3/4; (xviii) *y*+1, −*x*, *z*−3/4; (xix) −*y*+2, *x*, *z*−1/4; (xx) −*y*+1, *x*−1, *z*−1/4; (xxi) *x*, *y*, *z*−1; (xxii) −*x*+1, −*y*+1, *z*−1/2; (xxiii) −*x*, −*y*+1, *z*−1/2; (xxiv) −*x*+1, −*y*, *z*+1/2.
